# Transforming growth factor-β superfamily members as potential biomarkers for adolescent major depressive disorder

**DOI:** 10.3389/fpsyt.2025.1655332

**Published:** 2025-09-01

**Authors:** Xiaolan Wang, Yiting Kong, Jingyue Xiang, Zhenghao Jiang, Yijia Wang, Xiaorong Chen, Liyang Wan, Su Hong, Li Kuang

**Affiliations:** ^1^ Mental Health Center, University-Town Hospital of Chongqing Medical University, Chongqing, China; ^2^ Department of Psychiatric Center,The First Affiliated Hospital of Chongqing Medical University, Chongqing, China

**Keywords:** depression, TGF-β superfamily, biomarkers, adolescents, cross-sectional study

## Abstract

**Background:**

Growing evidence implicates the transforming growth factor-β (TGF-β) superfamily in neurodevelopment and immunoregulatory processes, with several members associated with depression in adults. However, the relationship between specific TGF-β superfamily members and adolescent major depressive disorder (MDD) remains poorly understood. This study aimed to evaluate whether specific TGF-β superfamily members could serve as biomarkers for adolescent MDD.

**Methods:**

In this cross-sectional study, 180 adolescents were enrolled,including individuals diagnosed with MDD and healthy controls (HC). Depressive symptoms were assessed using the 17-item Hamilton Depression Rating Scale (HAMD-17). Serum concentrations of transforming growth factor-β1 (TGF-β1),growth differentiation factor 11 (GDF11), and growth differentiation factor 15 (GDF15), were quantified via enzyme-linked immunosorbent assay (ELISA). Demographic and clinical characteristics were analyzed.Correlation and multiple linear regression analyses were performed to explore associations between serum TGF-β superfamily levels and depression severity. Furthermore, receiver operating characteristic (ROC) curve analysis was used to assess the diagnostic potential of these TGF-β superfamily members in MDD.

**Results:**

Compared with healthy controls, the MDD group exhibited significantly lower serum levels of TGF-β1 and GDF11,and higher levels of GDF15 (all *p*< 0.05). Correlation analysis revealed that serum TGF-β1 and GDF11 were negatively associated with depression severity, while GDF15 levels showed a positive correlation. All three molecules demonstrated strong diagnostic potential for MDD. Combination of these three proteins demonstrated much better diagnostic effectiveness.

**Conclusions:**

Serum TGF-β1, GDF11, and GDF15 levels may serve as promising biomarkers for adolescent MDD, offering potential utility in identifying disease susceptibility. These findings highlight the TGF-β superfamily’s role in adolescent depression and warrant further mechanistic investigation.

## 1 Introduction

Major depressive disorder (MDD) is a debilitating psychiatric condition characterized by core diagnostic criteria including persistent low mood lasting ≥2 weeks, marked anhedonia, and associated symptoms such as cognitive impairment and autonomic dysfunction (1–3). The high disability burden of this disorder warrants particular attention, especially among adolescents during critical neurodevelopmental periods.

Adolescence is characterized by unique neurobiological features, including enhanced synaptic plasticity and active neural circuit remodeling. These processes facilitate cognitive-emotional development but also heighten vulnerability to stress-induced psychiatric disorders such as MDD (4–7). Depression prevalence in children and adolescents has increased significantly, with recent epidemiological studies reporting rates of 5%-8% (8). Studies indicate that the one-year prevalence rate of depression during mid-to-late adolescence ranges from 2% to 8%, with nearly 20% of individuals reporting experiencing at least one depressive episode before the age of 18 (9, 10). These findings highlight the urgent need to identify reliable biomarkers that could enable early diagnosis and inform targeted therapeutic interventions.

Although the pathogenesis of depression is complex and not fully understood, in recent years, the two mechanisms of inflammation and neurogenesis have gradually become the focus of research. Inflammation plays a crucial role in depression pathogenesis. Evidence suggests that inflammatory processes may contribute to depressive symptoms by influencing oxidative stress, neurotransmitter balance, neuroendocrine function, and hippocampal neurogenesis (11, 12). Patients with severe depression frequently exhibit elevated inflammatory markers, including IL-1β, IL-6, TNF-α, and CRP (13–15), compared with healthy individuals (16–18). In addition to classical inflammatory factors, large-scale population studies have revealed other potential biomarkers for depression. For instance, two studies based on the National Health and Nutrition Examination Survey (NHANES) database found that elevated serum Klotho levels were significantly associated with depression risk, but Mendelian randomization did not confirm a causal relationship (19). Similarly, increased serum apolipoprotein B was also associated with depression risk, again lacking genetic evidence for causation. These markers may reflect disease state rather than causal factors (20). Neurogenesis refers to the process through which neural precursor cells differentiate to generate new, mature neurons that then integrate into an existing neural network to carry out their functions (21). The two primary sites of neurogenesis are the subventricular zone (SVZ) of the lateral ventricle and the dentate gyrus (DG) of the hippocampus (22). Increasing evidence indicates that disrupted hippocampal neurogenesis plays a crucial role in the development of depression in rodents (23–25). Given these research developments, there is an urgent need to further investigate peripheral biomarkers associated with depression. In this context, the transforming growth factor-β (TGF-β) family has garnered significant attention due to its diverse biological functions.

The TGF-β family is composed of over 30 structurally related polypeptide growth factors, such as activins, nodals, TGF-βs, bone morphogenetic proteins (BMPs), and growth and differentiation factors (GDFs) (26). TGF-β superfamily is ubiquitously expressed in mammalian systems and exhibits diverse biological activities. These proteins regulate fundamental cellular processes (proliferation, differentiation, migration, apoptosis) and play critical roles in embryogenesis, osteochondral development, immune-endocrine crosstalk, and tumorigenesis. Their roles in neuroprotection and neural repair following injury have also been well documented (27). In view of the complexity and diversity of the pathogenesis of depression and the richness of the biological functions of the TGF-β superfamily, more and more studies have revealed that the TGF-β superfamily may be involved in the pathophysiological process of depression. This involvement is primarily mediated through several core mechanisms, including modulating neuroplasticity, mediating neuroinflammatory responses, and modulating stress adaptation. This link implies that members of the TGF-β superfamily may be important participants in the onset and progression of depression. Therefore, the TGF-β superfamily is expected to be a potential biomarker of depression and may provide innovative avenues for diagnostic strategies and therapeutic interventions.

TGF-β1, a crucial member of the TGF-β superfamily, is primarily secreted by astrocytes and microglia. This cytokine plays critical roles in both immunomodulation and neuroprotection in the central nervous system (28). As an essential neurotrophic factor, TGF-β1 significantly contributes to hippocampal synaptic plasticity and memory formation. It exhibits distinct gene-environment interactions and participates in modulating pathways associated with stress response and depression pathogenesis. Notably, TGF-β1 may serve as a potential biomarker for predicting adult depression stemming from early-life adversities such as childhood maltreatment and significant stressful events (29, 30). Moreover, the expression level of TGF-β1 is significantly correlated with the severity of depression and cognitive dysfunction (28).

GDF15 is a critical stress-responsive protein, typically expressed at low levels in healthy individuals and young subjects. However, its expression increases significantly under various pathophysiological conditions, including inflammation, oxidative stress, hypoxia, and tissue damage (31). Functionally, GDF15 acts not only as a key regulator of systemic homeostasis but also plays a protective role during inflammatory responses. Furthermore, it serves as a reliable biomarker for oxidative stress and cellular senescence (32). These properties suggest that GDF15 may play an important role in the pathophysiology of stress-related mental disorders.

Particularly noteworthy is GDF11, which plays a crucial regulatory role in the central nervous system. Owing to its potential to modulate neurogenesis and cognitive function, GDF11 has recently emerged as a prominent focus in depression research (33). Preclinical studies have demonstrated that GDF11 is essential for the development of the central nervous system, particularly during brain formation (34). Furthermore, as a modulator of functional neuronal firing, GDF11 not only promotes neurogenesis and neuronal autophagy but also alleviates depression-like behaviors (33).

While numerous studies have suggested a potential association between the TGF-β superfamily and depression, the existing evidence remains inconsistent. For instance,one study reported that both suicidal and non-suicidal depressed patients exhibited significantly higher *in vitro* TGF-β1 secretion compared to healthy controls (35). In contrast, a study by Lee and Kim found no statistically significant differences in these measurements (36). More importantly, existing research has focused primarily on adult populations, leaving the expression patterns of TGF-β superfamily members in adolescents with depression largely unexplored. To address this gap, our study aims to systematically analyze the expression levels of TGF-β1, GDF11 and GDF15 in peripheral blood of adolescent patients with MDD, and to explore their correlations with depression severity. These efforts are expected to provide novel experimental evidence for elucidating the pathogenesis of adolescent depression.

## 2 Materials and methods

### 2.1 Study design and participants

In this study, we employed a rigorous approach to sample size calculation. Based on pre-experimental data revealing a difference in TGF-β1 levels between the depression group (7.78 ± 2.44 ng/ml) and the control group (9.81 ± 2.65 ng/ml), an initial effect size Cohen’s d of 0.797 was determined. In order to avoid the overestimation of the effect size that may be caused by the small sample pre-experiment, we conservatively adopted a medium effect size (d=0.5) for the formal sample size calculation. Utilizing GPower 3.1 software with parameters set at α=0.05 and test power of 80%, we calculated a theoretical sample size of 64 subjects per group. Taking into account an anticipated dropout rate of 20%, potential technical variability introduced by the simultaneous measurement of multiple biomarkers (TGF-β1, GDF11, and GDF15), and the necessity for covariate control, we prudently expanded our sample size. Consequently, we finalized the inclusion of 90 subjects in each group.

From October 2021 to July 2022,this study enrolled adolescents aged 13–17 years, comprising two groups: (1) patients with MDD were diagnosed at the First Affiliated Hospital of Chongqing Medical University, meeting the Diagnostic and Statistical Manual of Mental Disorders, Fifth Edition (DSM-5) diagnostic criteria as confirmed by two senior psychiatrists using the Mini International Neuropsychiatric Interview for Children and Adolescents (MINI-KID) (37). These patients had Hamilton Depression Rating Scale-17 (HAMD-17) scores of ≥17, were treatment-naïve, and were right-handed. (2) Healthy controls (HC) who were age-, gender-, and education-matched volunteers with HAMD-17 scores of <7, no personal or family history of psychiatric disorders, and no history of psychotropic medication use.Common exclusion criteria for both groups included: (1) the presence of severe systemic diseases such as cardiovascular, hepatic, renal, endocrine (e.g., thyroid dysfunction), hematologic, autoimmune, or other chronic inflammatory disorders; (2) current and past organic brain diseases, history of head trauma, or seizures; (3) comorbid mental disorders or substance use disorders; and (4) unwillingness to participate in the study.

All participants voluntarily took part in this study and had an education level of primary school or above. The study was approved by the Ethics Committee of the First Affiliated Hospital of Chongqing Medical University (2021–546), and written informed consent was obtained from all participants and their legal guardians.

### 2.2 Measures

#### 2.2.1 Demographic data

Demographic information,including name,age, gender and body mass index (BMI),were selected on the basis of clinical relevance and previous literature (38, 39). These variables were collected through a self-designed scale for this study. Although names were initially collected for administrative purposes, all personal identifiers, including names, were removed from the dataset before analysis to ensure participant anonymity and data privacy.

#### 2.2.2 Diagnosis of depression

The HAMD-17 is a widely used clinical tool for assessing the severity of depressive symptoms (40). This 17- item scale provides a quantitative evaluation of depressive severity, with lower scores indicating milder symptoms and higher scores reflecting more severe conditions.Specifically, the scoring system is categorized as follows: scores ≤7 are considered within the normal range; scores between 8 and 16 indicate mild depression; scores between 17 and 23 suggest moderate depression; and scores exceeding 24 are classified as severe depression (41).

#### 2.2.3 Determination of serum TGF-β superfamily members

In a fasting state, 5 milliliters of peripheral venous blood was collected from each participant into serum separator tubes. After incubation at room temperature for 20 minutes, the samples were centrifuged at 3,500 rpm for 15 minutes. The resulting supernatant was transferred into cryovials and stored at –80°C for subsequent analysis.Serum levels of TGF-β1, GDF11, and GDF15 were measured using enzyme-linked immunosorbent assay (ELISA) kits (Biosine Biological Products Company, Chongqing, China), following the manufacturer’s instructions.

### 2.3 Statistical analyses

We described the demographic and clinical characteristics of both the MDD group and healthy control group.Continuous variables were assessed for normality using the Kolmogorov-Smirnov test. Data with a normally distributed were presented as mean ± standard deviation and analyzed using independent samples t-tests, whereas non-normally distributed data were presented as median (interquartile range, IQR) and analyzed with Mann-Whitney U tests.

Categorical variables were presented as percentages and compared using chi-square tests. Bivariate correlations were examined using Spearman’s correlation analysis due to the non-normal distribution of most variables.Multiple linear regression models were constructed to identify predictors of depression severity, with relevant TGF-β superfamily members included as independent variables and age, sex, and BMI entered as covariates.The diagnostic utility of candidate biomarkers was evaluated using receiver operating characteristic (ROC) curve analysis. Sensitivity, specificity, positive predictive value (PPV), negative predictive value (NPV), and Youden’s index were calculated based on optimal cut-off thresholds determined from the ROC curves. All statistical analyses were performed with SPSS (version 25.0), GraphPad Prism (version 10.1.2), and R software (version 4.4.1). Statistical significance was set at a two-tailed p-value < 0.05.

## 3 Results

### 3.1 Demographic comparison between MDD and HC groups

A total of 180 participants were included in this study, comprising 90 patients with MDD and 90 matched HC. Comparative analysis revealed a statistically significant difference in HAMD-17 scores between the two groups (*p* < 0.001). However, no significant differences were observed in age, gender distribution, or BMI between the two groups (**Table 1**).

**Table 1:** Demographic and clinical characteristics of study participants.

Variables	MDD group (N=90)	HC group (N=90)	Z/χ^2^	P Value
Age	15.1 (14,16)	15.4 (15,16)	Z=-1.596	0.111
Gender			χ^2^ = 0.106	0.745
Male	26 (48.10%)	28 (51.90%)		
Female	64 (50.80%)	62 (49.20%)		
BMI	20.76 (17.92,23.25)	20.35 (18.19,21.46)	Z =-0.04	0.968
HAMD-17	23.13 (20,26)	1.11 (0,2)	Z=-11.689	<0.001

BMI, body mass index; HAMD-17, 17-item Hamilton Depression Scale.

### 3.2 Comparative analysis of TGF-β superfamily levels in MDD and HC groups

**Table 2** presents the group differences in serum levels of TGF-β superfamily proteins. Patients with MDD exhibited significantly higher concentrations of GDF15 compared to healthy controls (*p* < 0.001), while serum levels of TGF-β1 and GDF11 were significantly lower in the MDD group (both *p* < 0.001).

**Table 2:** Comparison of TGF-β famliy members between the two groups.

Variables	MDD group (N=90)	HC group (N=90)	t/Z	P Value
TGF-β1(ng/ml)	7.21 (5.88,8.79)	10.77 (9.71,12.21)	Z =-9.155	<0.001
GDF11(pg/ml)	296.89 ± 83.67	482.09 ± 75.79	t=15.563	<0.001
GDF15(pg/ml)	845.85 (730.23,989.23)	487.92 (391.98,576.48)	Z =-10.886	<0.001

TGF-β1,transforming growth factor-β1; GDF11, Growth Differentiation Factor 11; GDF15, Growth Differentiation Factor 15.

### 3.3 Correlations of TGF-β superfamily proteins with depression severity

As presented in **Table 3**, correlation analysis was conducted. Results indicated that demographic factors such as age and BMI were not significantly correlated with any of the other variables (all p>0.05). Regarding associations among the core biomarkers and depression severity, GDF11 was negatively correlated with GDF15 (r=-0.643, *p*<0.01) and HAMD-17 scores (r=-0.663, *p*<0.01), while showing a positive correlation with TGF-β1 (r=0.493, *p*<0.01). GDF15 was positively correlated with HAMD-17 scores (r=0.705, *p*<0.01), but negatively correlated with TGF-β1(r=-0.573, *p*<0.01). Additionally, TGF-β1 was negatively correlated with HAMD-17 scores (r=-0.646, *p*<0.01).

**Table 3:** Correlation between variables and the severity of depression (r).

Variables	Age	BMI	GDF11	GDF15	TGF-β1	HAMD-17 score
Age	1					
BMI	0.144	1				
GDF11	0.04	0.088	1			
GDF15	-0.091	-0.034	-0.643^**^	1		
TGF-β1	0.003	0.004	0.493^**^	-0.573^**^	1	
HAMD-17 score	-0.144	-0.051	-0.663^**^	0.705^**^	-0.646^**^	1

BMI, body mass index; GDF11, Growth Differentiation Factor 11; GDF15, Growth Differentiation Factor 15; TGF-β1, transforming growth factor-β1; HAMD-17, 17-item Hamilton Depression Scale; ** P<0.01.

### 3.4 Predictive relationship between TGF-β superfamily proteins levels and depressive symptom severity

We performed a multiple linear regression analysis,with results presented in **Table 4**. The model showed good fit with an adjusted R² of 0.744, well above the commonly accepted threshold of 0.2, confirming the reliability of the results. Additionally,all variance inflation factor (VIF) values were below 10, indicating no significant multicollinearity among the independent variables. After controlling for age, gender and BMI, the analysis revealed that TGF-β1 and GDF11 were significant negative predictor of depression severity (both *p <*0.001), while GDF15 showed significant positive predictive relationship (*p*<0.001). The final regression model was expressed by the equation: Depression severity = 29.260 − 1.254* TGF−β1− 0.032* GDF11 + 0.02* GDF15 .

**Table 4:** Regression analysis of depression severity predictors.

	B	Sb	Beta	t	p	VIF	R^2^	F
TGF-β1	-1.254	0.203	-0.289	-6.171	<0.001	1.527	0.744	87.538
GDF11	-0.032	0.005	-0.344	-6.925	<0.001	1.727		
GDF15	0.02	0.003	0.382	7.27	<0.001	1.932		

TGF-β1, transforming growth factor-β1; GDF11, Growth Differentiation Factor 11; GDF15, Growth Differentiation Factor 15.

### 3.5 Assessment of the diagnostic performance of TGF-β superfamily proteins

**Figure 1:**
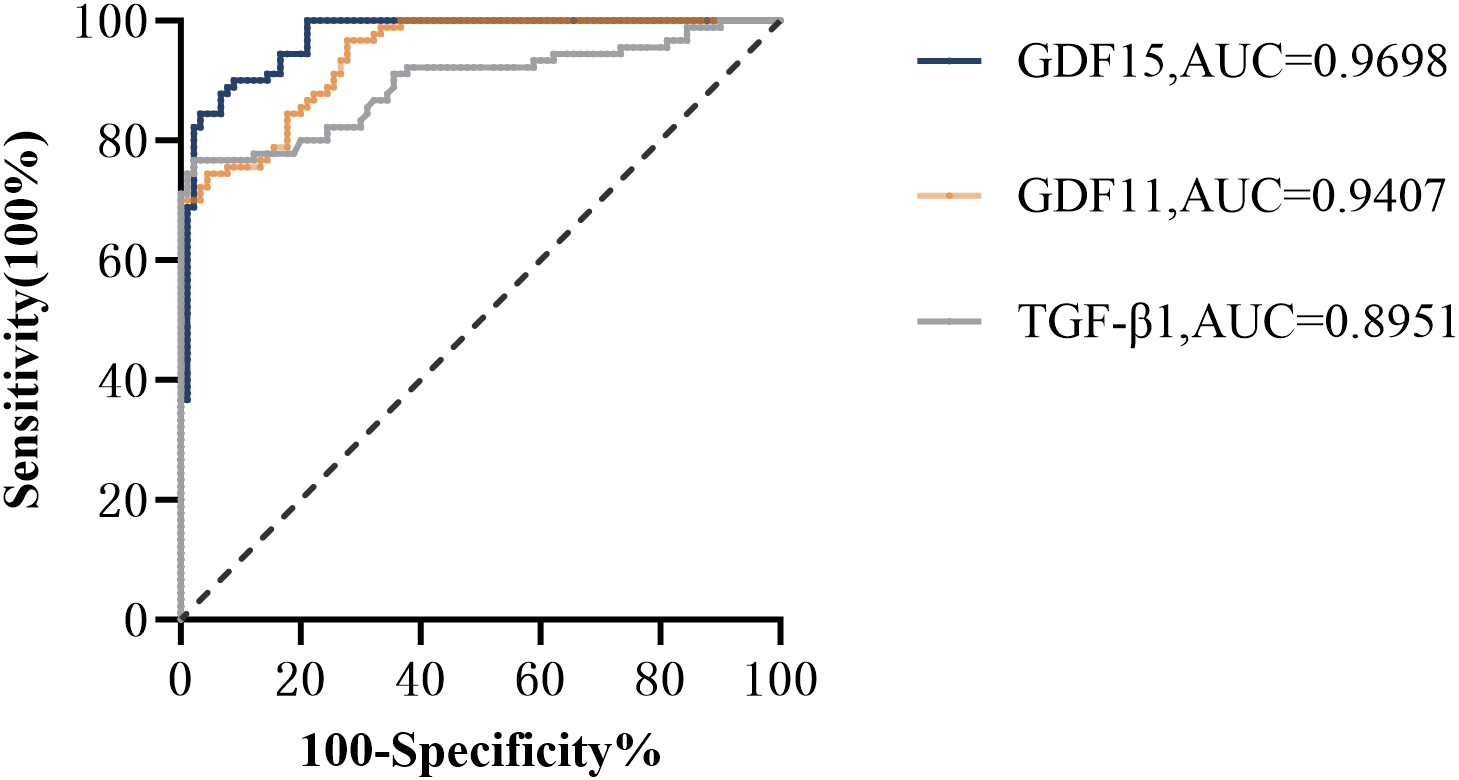
Receiver operator characteristic (ROC) analysis of TGF-β superfamily members for prediction of depression. GDF15, Growth Differentiation Factor 15; GDF11, Growth Differentiation Factor 11; TGF-β1, transforming growth factor-β1.

**Figure 2:**
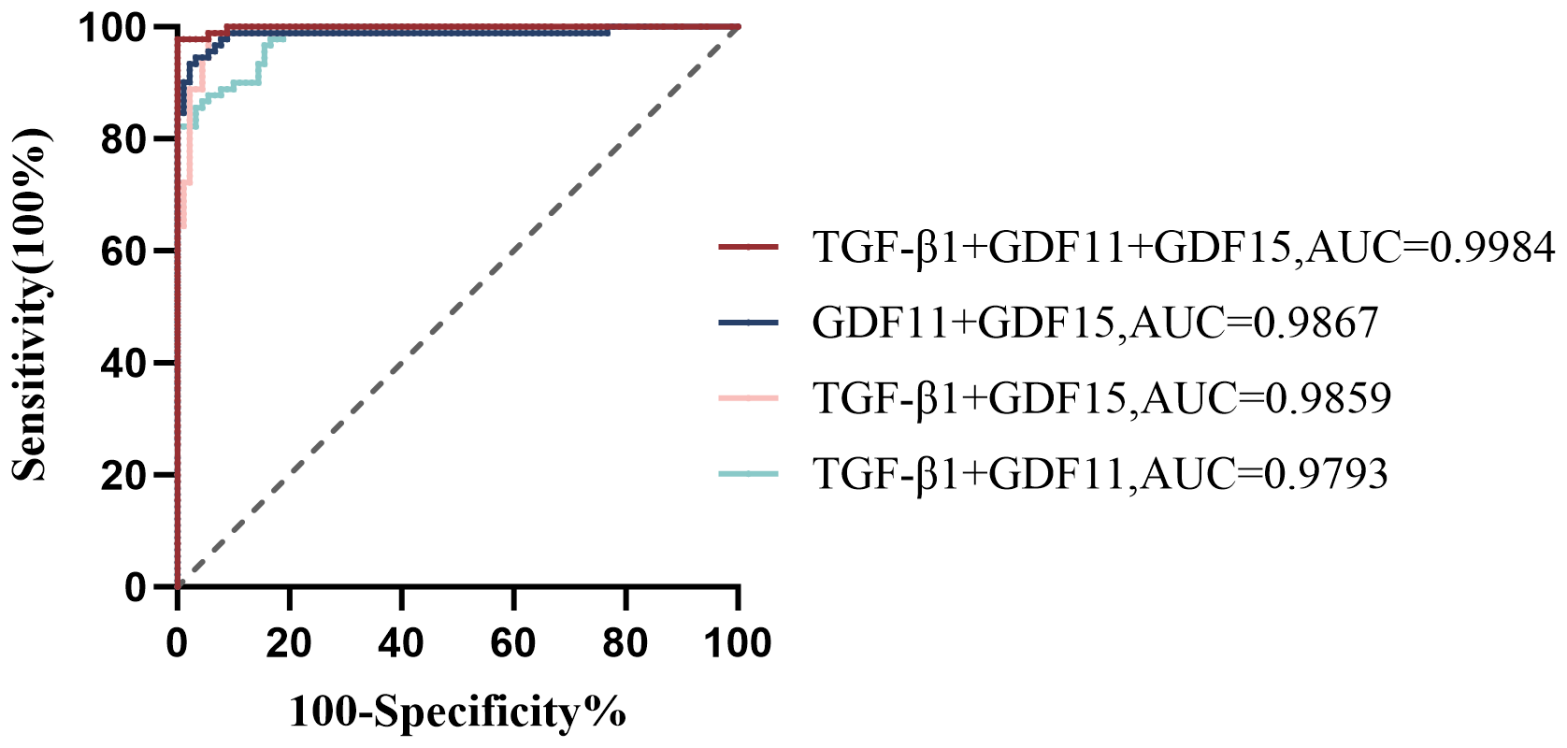
Receiver operator characteristic (ROC) analysis of TGF-β1, GDF11, and GDF15 combinations for predicting depression. TGF-β1, transforming growth factor-β1; GDF11, Growth Differentiation Factor 11; GDF15, Growth Differentiation Factor 15.

**Figure 3:**
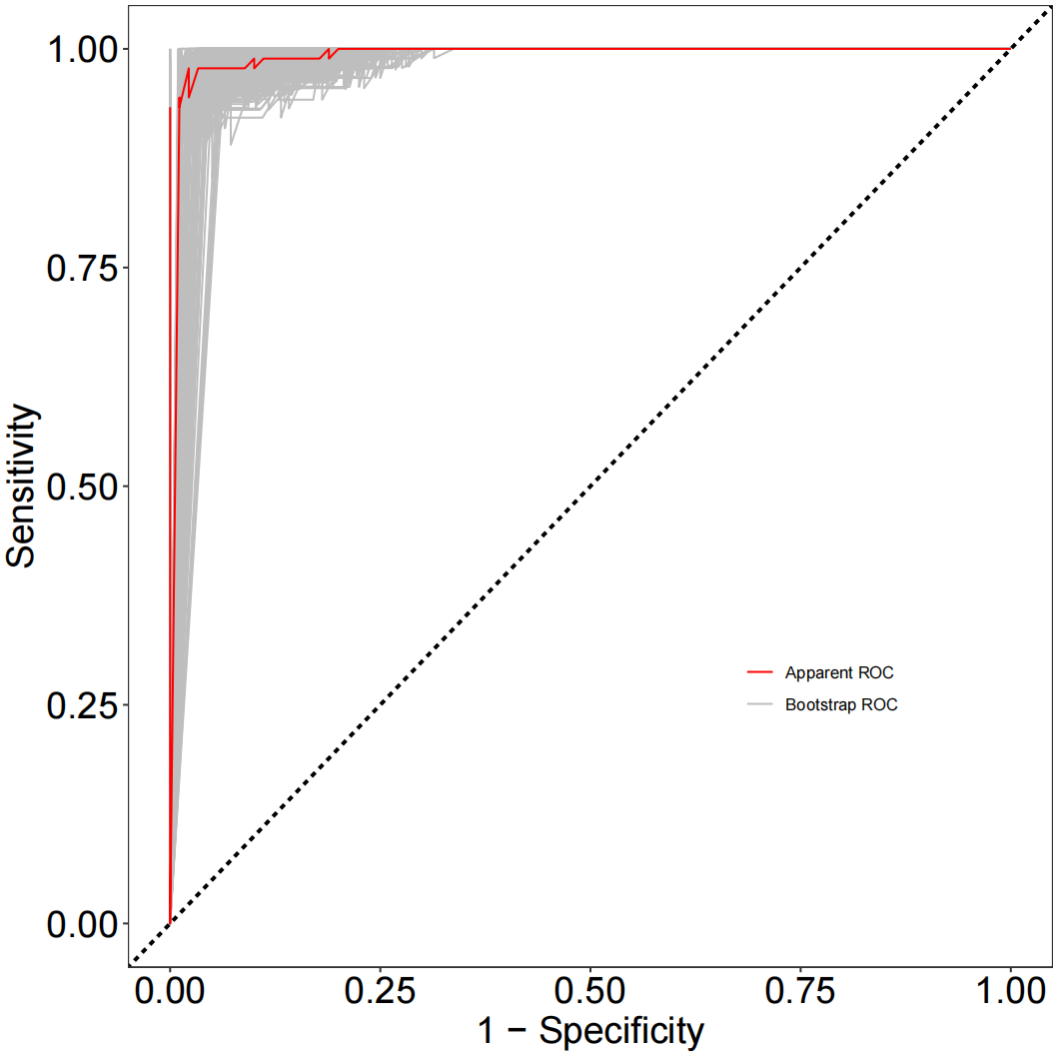
Bootstrap receiver-operating characteristic curve for the three-marker panel.

As depicted in **Figure 1**, receiver operating characteristic (ROC) curve analysis was conducted to evaluate the predictive power of TGF-β superfamily members in adolescents with MDD. The results revealed that GDF15 possessed the most outstanding discriminative ability for predicting depression, with an area under the curve (AUC) of 0.9698 [95% CI (0.9488-0.9907), *p*< 0.0001]. This performance surpassed that of GDF11 [AUC =0.9407, 95% CI (0.9110-0.9705), *p*< 0.0001] and TGF-β1 [AUC =0.8951, 95% CI (0.8462-0.9440), *p*< 0.0001]. As shown in **Figure 2**, the combination of TGF-β1, GDF11, and GDF15 demonstrated the highest diagnostic power, with an AUC of 0.9984[95% CI(0.9957-1.000), *p*<0.0001]. Other combinations also showed significant performance: GDF11 + GDF15 reached an AUC of 0.9867 [95% CI(0.9694-1.000), *p* < 0.0001], whereas TGF-β1 + GDF15 yielded an AUC of 0.9859 [95% CI(0.9720-0.9998),*p* < 0.0001], and TGF-β1 + GDF11 achieved 0.9793 [95% CI(0.9646-0.9939), *p* < 0.0001]. **Table 5** summarizes the corresponding sensitivity, specificity,PPV,NPV, and Youden’s index for each single marker and their combinations.To further assess the reliability and generalizability of the three-marker combination model,we conducted bootstrap validation with 500 resampling iterations to further evaluate model robustness. The bias-corrected AUC from bootstrap analysis was 0.9909 (95% CI: 0.9826–0.9992) (**Figure 3**), confirming the stability and reliability of our findings.

**Table 5:** Diagnostic performance of individual and combined TGF-β1, GDF11, and GDF15 for MDD.

Variables	Sensitivity	Specificity	PPV	NPV	Youden’s index
TGF-β1	0.7667	0.9778	0.9718	0.8073	0.7445
GDF11	0.9556	0.7444	0.7890	0.9437	0.7000
GDF15	0.9667	0.8444	0.8614	0.9620	0.8111
TGF-β1+GDF11	0.9667	0.9111	0.9158	0.9647	0.8778
TGF-β1+GDF15	0.9222	0.9444	0.9432	0.9239	0.8666
GDF11+GDF15	0.9889	0.9444	0.9468	0.9884	0.9333
TGF-β1+GDF11+GDF15	0.9778	0.9778	0.9778	0.9778	0.9556

TGF-β1, transforming growth factor-β1; GDF11, Growth Differentiation Factor 11; GDF15, Growth Differentiation Factor 15; PPV, Positive Predictive Value; NPV, Negative Predictive Value.

## 4 Discussion

In this cross-sectional study, we assessed serum levels of key members of the TGF-β superfamily in adolescents with MDD and healthy controls, and explored their associations with clinical features and depression severity. As far as we know, this is the first study to specifically focus on adolescent populations in this context.Our findings suggest that TGF-β superfamily members- particularly GDF15, TGF-β1, and GDF11-may play important roles in the pathophysiology of depression. Notably, GDF15 levels were significantly elevated in patients with MDD, while TGF-β1 and GDF11 levels were significantly reduced. These markers also demonstrated strong correlations with depression severity: GDF15 was positively associated, while TGF-β1 and GDF11 were negatively correlated.

Our findings demonstrated significantly elevated circulating GDF15 levels in MDD patients compared to healthy controls, which is consistent with previous reports (42–44). Additionally, we observed markedly reduced peripheral serum levels of both TGF-β1 and GDF11 in the MDD group, aligning with findings from other studies that also reported substantial decreases in these proteins among individuals with depression (28, 33, 45). However, inconsistencies persist in the current literature: While Lee et al. reported no significant differences in blood TGF-β1 levels between MDD patients and controls, their study was limited by a relatively small sample size (n=60 total participants), potentially compromising statistical power (36). In contrast, Kim et al. documented increased TGF-β1 levels in MDD patients (46). Notably, their research exclusively comprised hospitalized individuals who typically present with more severe symptomatology and clinical severity.These discrepancies may be attributed to differences in the study populations and sample sizes. Collectively, these findings highlight the necessity for large-scale, rigorously designed studies to clarify the role of TGF-β1 in depression and to address inconsistencies in the existing literature.

This study found that the dysregulation of TGF-β superfamily members in patients with depression may reflect their roles in neuroinflammation, neurogenesis, and synaptic plasticity, all of which are closely related to the pathophysiology of depression (23, 25, 47–50). At the molecular level, GDF15 is a stress-responsive cytokine secreted by multiple cells, including macrophages, endothelial cells, and neurons, in response to oxidative stress, injury, and inflammation (51, 52). Given its role as a pro-inflammatory stress signal mediating inflammatory responses, elevated GDF15 concentrations may be associated with “inflammaging,” a chronic low-grade inflammation that contributes to aging and disease processes. Inflammation is a crucial role in the pathogenesis of depression, with evidence suggesting that it influences oxidative stress, neurotransmitter balance, neuroendocrine function, and hippocampal neurogenesis, thereby contributing to the development of depressive symptoms (11, 12). Additionally, GDF15 has also been reported to promote astrocyte remodeling and strengthen tight junction at the blood–brain barrier (BBB), which is closely associated with the occurrence and development of depression (53). In the present study, correlation analysis revealed a positive association between GDF15 levels and depression severity, consistent with previous findings (54). Collectively, these results suggest that elevated GDF15 may reflect an overactive inflammatory response and impaired BBB homeostasis, ultimately contributing to the development and progression of depression.

GDF11 is a key regulator involved in bone formation, skin regeneration, neurogenesis, inflammatory processes, and tumorigenesis (55). In addition, GDF11 has been shown to regulate the functional firing of neurons, promote neurogenesis and neurophagy, thereby alleviating depressive-like behaviors (33). Evidence indicates that weakened GDF11 signaling increases the risk of depression by enhancing the inflammatory response and increasing astrocyte activity (56, 57). These findings collectively suggest that decreased GDF11 levels may indicate impaired anti-inflammatory signals or inadequate neurotrophic support, ultimately leading to the development of depression.

TGF-β1 is a multifunctional cytokine that plays a vital role in regulating neuroplasticity, including synaptic formation and maintenance, neuronal survival and differentiation, and BBB integrity (58). The decrease in serum levels of TGF-β1 in patients with depression may reflect the dual deficiency in neuroprotective and immunomodulatory functions. As a multifunctional neurotrophic factor, reduced TGF-β1 weakens its promotion of synaptic plasticity, reduces neuronal viability, and disrupts BBB integrity (59). At the same time, the weakening of its anti-inflammatory activity may lead to microglial overactivation and increased release of pro-inflammatory cytokines such as IL-6, TNF-α, thereby exacerbating the neuroinflammatory response (60). These changes are consistent with the hypothesis of impaired neuroplasticity and neuroinflammation in depression. Clinical observations have shown that TGF-β1 levels are inversely correlated with the severity of depressive symptoms (61), and a resurgence of TGF-β1 levels can be observed after treatment with some antidepressants (36, 62), suggesting that TGF-β1 may not only participate in the pathophysiological process of depression but also serve as a potential biomarker of treatment response. Notably, both TGF-β1 and GDF11 are inversely correlated with depression severity, which is consistent with previous studies (33, 61), further supporting their potential role as a moderator of disease progression.

Recent studies have identified High Mobility Group Box 1 (HMGB1) as a key mediator of neuroinflammation in depression. HMGB1 is actively released from neurons and glial cells under conditions of stress or injury, once released, HMGB1 binds to pattern recognition receptors, including Toll-like receptors (TLRs) and receptors for advanced glycation end products (RAGE). These interactions potently activate NF-κB signaling pathways, ultimately driving the production of pro-inflammatory cytokines including IL-1β and TNF-α (63). These HMGB1-driven inflammatory cascades may interact with the dysregulation of TGF-β superfamily members observed in our study. For instance, elevated GDF15 may exacerbate HMGB1-mediated inflammation and blood–brain barrier BBB disruption, whereas reduced levels of GDF11 and TGF-β1 may reflect impaired anti-inflammatory and neurotrophic signaling. Collectively, these pathways may synergistically contribute to neuroinflammation, synaptic dysfunction, and the pathogenesis of depression. However, the precise crosstalk between HMGB1 signaling and the TGF-β superfamily remains to be elucidated and warrants further investigation.

Compared to standalone biomarkers, a combinatorial biomarker panel may reduce inter-population and inter-subgroup variability, thereby improving diagnostic precision. Accumulating evidence has demonstrated that multi-analyte profiling significantly outperforms single-marker approaches in diagnostic efficacy (64, 65). Consistent with these observations, our current findings revealed that serum levels of TGF-β1, GDF15, and GDF11 each exhibited considerable diagnostic accuracy for the detection of MDD when assessed as individual biomarkers. Additionally, our results show that the combination of these three proteins demonstrated much better diagnostic effectiveness. These findings suggest that serum levels of TGF-β1,GDF11,GDF15 all should be considered as a biomarker for MDD diagnosis, and their combination may serve as a promising multi-analyte diagnostic panel for MDD detection. Despite the excellent internal validation results, we acknowledge that these near-perfect AUC values warrant cautious interpretation. Sample selection bias, or lack of external validation may inflate performance estimates. Therefore, external validation in independent, multicenter cohorts is essential to confirm the generalizability and clinical utility of the proposed biomarker panel.

Given that the sample collection for this study was conducted during the COVID-19 pandemic, it is important to consider the broader psychosocial context in which these data were obtained. Research has shown that psychological distress and sleep problems increased markedly during this period (66, 67). For example, a large multicenter cross-sectional study conducted by Zhang et al. during the return-to-work phase in China reported that 18.3% of 36,795 participants experienced anxiety symptoms, 14.9% reported depressive symptoms, and 17.9% reported insomnia, underscoring the pandemic’s immediate and widespread impact on mental health. In the context of pandemic-related limitations on face-to-face assessments and strained healthcare resources, peripheral blood-based biomarkers hold potential as complementary tools for remote or large-scale screening, providing quantifiable reference data to prioritize mental health interventions and monitor treatment efficacy.The diagnosis of depression primarily relies on patient self-report and clinical interviews conducted by physicians, with a notable lack of objective biomarkers. While subjective assessment remains central to current diagnostic practices, it carries the risk of diagnostic heterogeneity, misdiagnosis, and missed diagnosis. This study provides compelling evidence for the dysregulation of TGF-β superfamily members in depression and highlights the potential of GDF15, TGF-β1, and GDF11 as promising biomarkers for both diagnosis and severity assessment. Future research should aim to evaluate the predictive value of these biomarkers in monitoring treatment response and tracking disease progression.Understanding the correlation between changes in GDF15, TGF-β1, and GDF11 levels and therapeutic outcomes may contribute to the development of individualized treatment strategies for depression. In addition, longitudinal studies are needed to investigate the temporal dynamics of these biomarkers and their relationship with the onset, duration, and recurrence of depressive episodes. In addition, by exploring the underlying molecular mechanisms of these TGF-β superfamily members on the pathogenesis of depression, valuable insights will be provided for the development of new therapeutic targets.

Several limitations should be acknowledged in this study. First, the cross-sectional design inherently limits causal inference and cannot exclude potential reverse causality. Therefore, future longitudinal or interventional studies are necessary to establish temporal relationships and assess whether TGF-β superfamily proteins can predict treatment response or relapse risk.Second, the exclusive focus on Han Chinese adolescents from a single geographic region may limit the generalizability of the findings to other ethnicities or populations. Third, although we matched participants by age, gender, and education level, and adjusted for several potential covariates, residual confounding from unmeasured factors, such as dietary patterns and physical activity, which may influence the outcomes cannot be entirely excluded. Fourth, despite excluding individuals with systemic inflammatory diseases, peripheral inflammatory markers such as CRP and IL-6 were not directly measured. These biomarkers may influence cytokine levels, and future studies should incorporate them to better control for inflammation-related confounding effects. Fifth, this study measured only serum levels of TGF-β1, GDF11, and GDF15 within the TGF-β superfamily. Other important family members, such as BMPs and Activins, were not assessed, potentially overlooking critical biological insights. Sixth, it should be noted that ELISA-based quantification may be influenced by several technical limitations, including cross-reactivity, matrix effects, and variations in sample handling or assay conditions. Additionally, sensitivity and specificity may vary depending on antibody quality and assay design.Although GDF11 and GDF15 show promise as candidate biomarkers, the ELISA kits currently used to quantify these proteins are certified for research use only and lack regulatory approval for clinical diagnostics. Consequently, their integration into routine clinical workflows remains restricted. Moreover,our study did not directly compare the clinical utility of GDF11 and GDF15 with existing biomarkers, such as serum CRP. Future research should incorporate such comparisons to ascertain the relative advantages of these new biomarkers. Seventh,peripheral serum measurements may not accurately reflect central nervous system expression patterns or activity states, which constrains the mechanistic interpretation of our findings. Eighth, the relatively small sample size in our study highlights the necessity for larger-scale replication studies to substantiate the diagnostic efficacy of the biomarkers under investigation. Future research with an expanded sample size should be designed to incorporate multiple comparison corrections, thereby ensuring sufficient statistical power to identify significant effects.In addition, although we performed internal validation using bootstrap methods, this approach cannot fully replace external validation in independent cohorts. External validation is essential to evaluate the generalizability and clinical applicability of the model. Future studies should validate the model in multicenter populations from diverse regions, ethnicities, and clinical backgrounds to confirm its universality. Finally, despite *a priori* sample size calculations ensuring adequate statistical power, the extremely high AUC values (near 1.0) observed in our model may reflect selection bias within the study population. Although the model demonstrates excellent statistical performance, its biological mechanisms and clinical interpretability require further exploration.

## 5 Conclusion

In summary, our study suggests that TGF-β superfamily members may serve as potential biomarkers for depression. Their observed dysregulation could contribute to depression-related neurobiological pathways, though mechanistic confirmation requires further investigation. These findings support the development of more objective diagnostic approaches for MDD while providing new perspectives on its pathophysiology. However, it is important to note that our results are preliminary and require further validation through additional studies.

## Data Availability

The raw data supporting the conclusions of this article will be made available by the authors, without undue reservation.
