# Supplementary material for: Transforming growth factor-β superfamily members as potential biomarkers for adolescent major depressive disorder
Source: Front Psychiatry. 2025 Sep 1;16:1655332. doi: 10.3389/fpsyt.2025.1655332 (PMC12433968; doi:10.3389/fpsyt.2025.1655332)
Supplement: Supplementary file 1 [file SupplementaryFile1.zip › Supplementary material/Supplementary materials.docx]

**Diagnostic Performance of Demographic Variables**

As shown in Supplementary Figure S1, the receiver operating characteristic (ROC) analysis revealed limited predictive power for age, sex, and BMI in depression risk assessment. The area under the curve (AUC) values with 95% confidence intervals (CIs) and statistical significance were as follows: age (AUC = 0.5667, 95% CI: 0.4788–0.6546, *p*=0.1224), gender (AUC = 0.5111, 95% CI: 0.4266–0.5957, *p*=0.7968), and BMI (AUC = 0.5015, 95% CI: 0.4161–0.5870,*p*=0.9715). These results suggest that none of these variables alone provides meaningful discriminatory ability for depression prediction.

**Figure S1** Receiver operator characteristic (ROC) curves of age, sex, and BMI for predicting depression


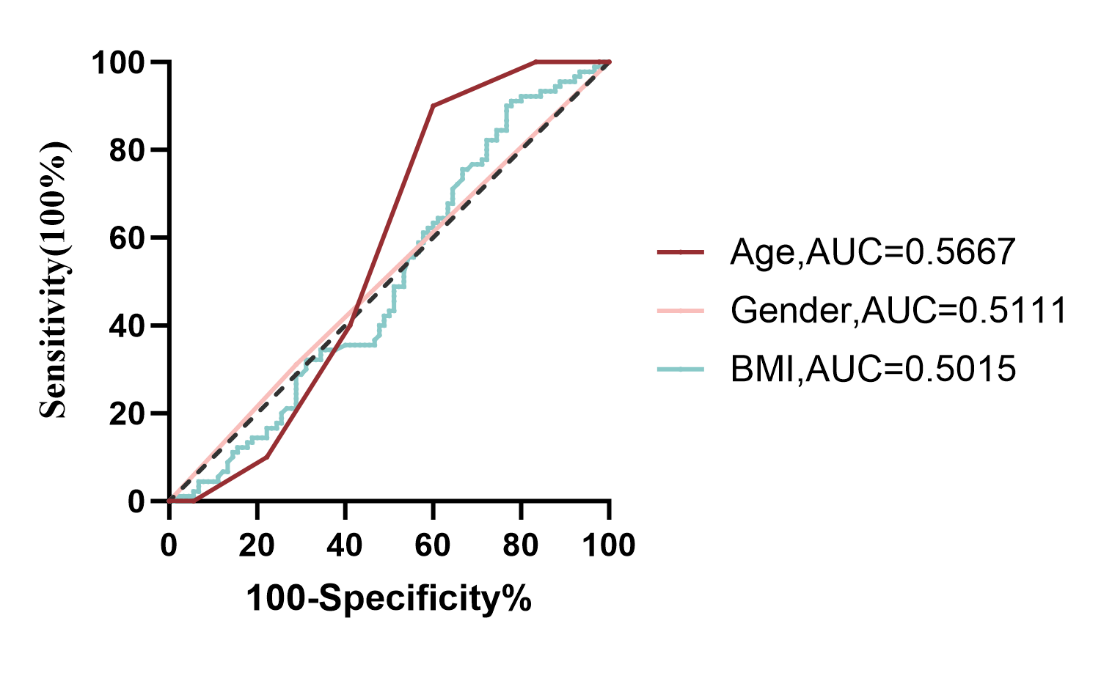


Abbreviations: BMI,body mass index
