# Supplementary figures and images for: Transforming growth factor-β superfamily members as potential biomarkers for adolescent major depressive disorder
Source: Front Psychiatry. 2025 Sep 1;16:1655332. doi: 10.3389/fpsyt.2025.1655332 (PMC12433968; doi:10.3389/fpsyt.2025.1655332)

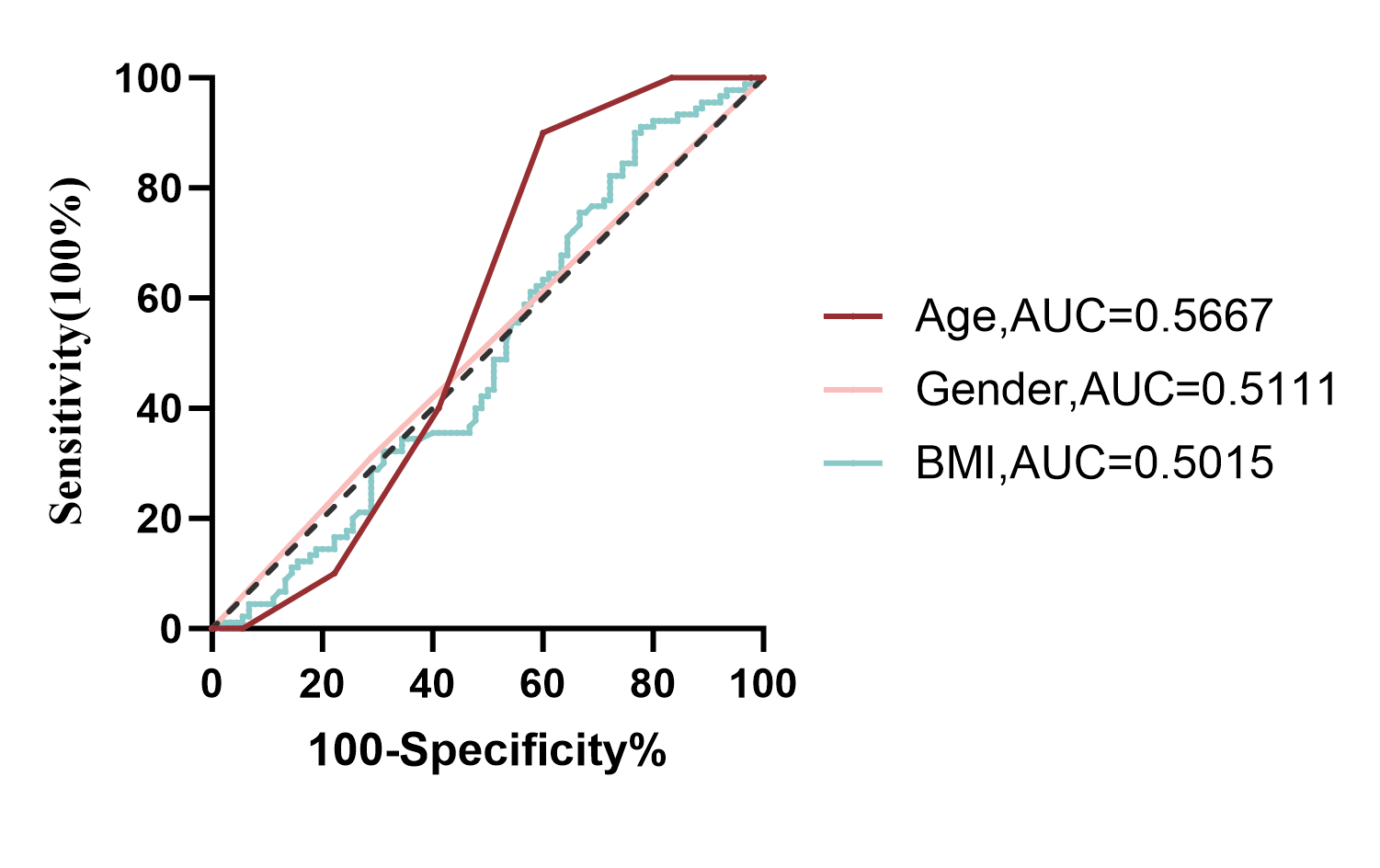

Supplement: Supplementary file 1 [file SupplementaryFile1.zip › Supplementary material/Figure S1.tif]
